# An Investigation into Which Methods Best Explain Children’s Exposure to Traffic-Related Air Pollution

**DOI:** 10.3390/toxics10060284

**Published:** 2022-05-26

**Authors:** Keith Van Ryswyk, Amanda J. Wheeler, Alice Grgicak-Mannion, Xiaohong Xu, Jason Curran, Gianni Caravaggio, Ajae Hall, Penny MacDonald, Jeffrey R. Brook

**Affiliations:** 1 Water and Air Quality Bureau, Health Canada, Ottawa, ON K1A 0K9, Canada; keith.vanryswyk@canada.ca (K.V.R.); amanda.wheeler@acu.edu.au (A.J.W.); 2 Mary MacKillop Institute for Health Research, Australian Catholic University, Melbourne, VIC 3000, Australia; 3 Menzies Institute for Medical Research, University of Tasmania, Hobart, TAS 7000, Australia; 4 Great Lakes Institute, University of Windsor, Windsor, ON N9B 3P4, Canada; grgica3@uwindsor.ca; 5 Department of Civil and Environmental Engineering, University of Windsor, Windsor, ON N9B 3P4, Canada; xxu@uwindsor.ca; 6 School of Population and Public Health, University of British Columbia, Vancouver, BC V6T 1Z8, Canada; jasonhkcurran@gmail.com; 7 Natural Resources Canada, 1 Haanel Drive, Ottawa, ON K1A 1M1, Canada; gianni.caravaggio@nrcan-rncan.gc.ca (G.C.); ajae.hall@nrcan-rncan.gc.ca (A.H.); penny.macdonald@nrcan-rncan.gc.ca (P.M.); 8 School of Public Health, University of Toronto, Toronto, ON M5T 3M7, Canada

**Keywords:** personal exposure, air pollution, traffic, hopanes

## Abstract

There have been several methods employed to quantify individual-level exposure to ambient traffic-related air pollutants (TRAP). These include an individual’s residential proximity to roads, measurement of individual pollutants as surrogates or markers, as well as dispersion and land use regression (LUR) models. Hopanes are organic compounds still commonly found on ambient particulate matter and are specific markers of combustion engine primary emissions, but they have not been previously used in personal exposure studies. In this paper, children’s personal exposures to TRAP were evaluated using hopanes determined from weekly integrated filters collected as part of a personal exposure study in Windsor, Canada. These hopane measurements were used to evaluate how well other commonly used proxies of exposure to TRAP performed. Several of the LUR exposure estimates for a range of air pollutants were associated with the children’s summer personal hopane exposures (r = 0.41–0.74). However, all personal hopane exposures in summer were more strongly associated with the length of major roadways within 500 m of their homes. In contrast, metrics of major roadways and LUR estimates were poorly correlated with any winter personal hopanes. Our findings suggest that available TRAP exposure indicators have the potential for exposure misclassification in winter vs. summer and more so for LUR than for metrics of major road density. As such, limitations are evident when using traditional proxy methods for assigning traffic exposures and these may be especially important when attempting to assign exposures for children’s key growth and developmental windows. If long-term chronic exposures are being estimated, our data suggest that measures of major road lengths in proximity to homes are a more-specific approach for assigning personal TRAP exposures.

## 1. Introduction

There is a considerable amount of research linking traffic-related air pollution (TRAP) with adverse health outcomes, including wheezing, incident asthma, birth outcomes, breast cancer, dementia, and cardiopulmonary morbidity and mortality [1,2,3,4,5,6,7,8,9,10,11,12,13]. A number of methods have typically been employed to characterize exposure to ambient TRAP for epidemiological studies. These include exposure assignment based on the individual’s residential proximity to roads, traffic density within set distances of the home, workplace or school, the use of individual pollutants as surrogates or markers (e.g., nitrogen dioxide (NO_2_), black carbon (BC)), dispersion models, and land use regression (LUR) models [14,15]. Some key limitations of exposure methods available for epidemiological research are the lack of data on individual’s location–activity patterns, along with access to high-resolution traffic intensity data, which leads to exposure misclassification. There is also the inability to identify the specific component(s) of traffic emissions (e.g., certain particle constituents or sizes and/or gaseous pollutants) or vehicle classes (e.g., gasoline, diesel) that are responsible for or have the greatest effects on health due to their lack of specificity, meaning they generally serve as broad TRAP indicators that are often inter-correlated.

Some of the most commonly employed markers of TRAP used in both ambient and personal monitoring include carbon monoxide, oxides of nitrogen (NOx), benzene, ultrafine particle counts (UFPs) and fine particle elemental carbon (EC) or BC [14]. However, while some of these markers may be predominantly from motor vehicle emissions in urban areas, none are purely unique to TRAP and could result from other common combustion sources. There are also a wide range of trace organic compounds in TRAP, which occur in both the gas and particle phase. These include aldehydes, alkenes, alkanes, aromatic hydrocarbons, polycyclic aromatic hydrocarbons (PAHs), nitro-PAHs, and heterocyclic compounds [16,17,18,19,20,21,22]. Most of these are also not unique to traffic emissions; however, one class of organics, referred to here as hopanes, is largely considered to be a unique marker of particles associated with TRAP. Hopanes are not found in gasoline and diesel fuel because they are in the higher boiling fraction of petroleum, but they are present in the engine oil lubricants used in all internal combustion engines [20,23,24,25,26,27,28,29]. While they can also be sourced from coal combustion, this source results in a hopane profile that is distinct from traffic-sourced hopanes. Previous research has identified coal and oil-specific ratios between two common hopanes (17α(H)-22, 29, 30-Trisnorhopane and 17α(H), 21β(H)-30-Norhopane), being 0.5–1.0 and 1.4–7.1, respectively [30]. Hopanes are also advantageous as TRAP markers because of their relative stability and relatively low volatility in the atmosphere compared to other organics [31,32,33]. However, they are not inert tracers given that they can photochemically degrade and return to the gas phase, depending upon temperature range and species [34,35].

Brook et al. showed that hopanes were prevalent in Canadian locations, including a highway tunnel [16]. They also demonstrated that hopane spatial patterns were related to proximity to traffic and were well correlated with intra-urban variations in NO_2_ [15]. Given their link to vehicle emissions, we hypothesize that, in an urban area, individual-level hopane measurements (i.e., personal exposures) are a specific marker of exposure to primary traffic emissions and they can be used to evaluate the benefits of using other proxy methods for estimating TRAP exposure applied in epidemiological research.

Individual-level exposure data can be obtained through the use of personal environmental monitors (PEM) [36,37,38]. Key factors that influence personal exposures to TRAP include living near roads and spending time near roads, how leaky residences are, which allows ambient air to penetrate indoors, and an individual’s time spent in transit [39,40,41,42]. Recent travel data showed that children aged 5–11 years spent on average 49 min a day in a vehicle [42]. Individuals are often exposed to the highest concentrations of TRAP when traveling in or near vehicles and can receive up to half of their share of daily exposure to TRAP from this microenvironment [14,41].

This paper takes a unique approach by comparing personal hopane exposure measurements with several metrics commonly employed to characterize exposure to TRAP in epidemiological research (e.g., proximity to roads and land-use regression estimates). These data were collected as part of a larger health and exposure study of children with asthma living in Windsor, ON, Canada. The Windsor study has included a range of exposure methods, which allows us to evaluate and compare such comparisons [37]. Our primary hypothesis is that the other commonly applied estimates of TRAP exposure derived for each child will exhibit varying degrees of correlation with personal exposures to hopanes, thus, providing unique insight into their true ability to serve as specific TRAP exposure indicators in epidemiological research.

## 2. Materials and Methods

In 2006, Health Canada and the University of Windsor conducted a personal exposure study in Windsor, ON, Canada. Forty-eight children with asthma (ages 10–13 years) participated in the personal monitoring study. Personal, residential indoor and outdoor air pollution exposures were assessed over a period of 10 days (typically Tuesday to Saturday), with a total of 5 continuous sampling days each in the winter (January–March) and summer (July–August). Only children with complete data collection over the 5-day period were included in the analyses of TRAP exposure discussed in this paper. A total of 17 personal winter and 25 summer samples were available for analysis, representing 210 exposure days in total.

### 2.1. Personal PM_2.5_ Monitoring and Sampling Methods

The personal monitoring included a personal DataRAM (pDR) (Thermo Scientific, Waltham, MA, USA) to measure continuous PM_2.5_ over each 5-day period. A pre-fired 37 mm quartz fibre filter (Pall-Gelman, Missisauga, ON, Canada) was placed in line after the pDR to collect the pre-separated PM_2.5_ samples that passed through the pDR inlet. The filter was in place for the entire 5 days of each season operating at a flow rate of 1.8 L per minute (lpm). The equipment, approximately 2 kg in weight, was contained within a backpack with the PEM located on the shoulder strap within the breathing zone and participants were asked to carry it with them wherever they went, leaving the backpack close to them to capture their exposure to air pollution when they were not able to carry it, such as during play, showering, and sleep.

### 2.2. Laboratory Analyses of Hopanes

The 5-day personal PM_2.5_ quartz fibre filters were analyzed for five hopanes; these were selected based on the laboratory’s analytical performance using direct thermal desorption gas chromatography/mass spectrometry (DTD-GC-MS) following the method detailed in Graham et al. [43]. For determination of personal hopane concentrations, two 7 mm diameter punches from the quartz filters (77 mm^2^ total area) were placed in Gerstel thermal desorption tubes, spiked with ββ-Hopane as the recovery standard, and desorbed at 335 °C for 10 min. In addition to the 30m DB-5MS column indicated in Graham et al. [43] we added a 10m guard column to improve peak shape. The hopanes reported here are: H17a, (17α(H)-22, 29, 30-Trisnorhopane); a_b_nor, (17α(H), 21β(H)-30-Norhopane); a_b_hop, (17α(H), 21β(H)-Hopane); H22S, (17α(H), 21β(H)-22S-Homohopane); and H22R, (17α(H), 21β(H)-22R-Homohopane). Final concentrations were recovery corrected on a sample-by-sample basis. Four sets of duplicate analysis were conducted and these paired samples were within 20 percent for H17a, a_b_Hop and a_b_nor (average percent difference of 10%). Results of duplicate analysis for H22S and H22R were less precise, with percent differences ranging from 2 to 45 percent (average of 27.5%).

### 2.3. Proxy Methods for TRAP Exposure

Proxy metrics for TRAP exposure included LUR estimates, NO_2_ concentrations sampled in participant homes, backyards, and on their person, measurements of PM_2.5_ and NO_2_ from a national air monitoring network, and metrics of local and major road networks.

The LUR models developed for Windsor over a three-year period have previously been described [44,45]. Pollutants included NO_2_, benzene, toluene, and PM_2.5_. Like many other LUR models, these were selected to capture exposures to a range of different sources of air pollution found in Windsor, including traffic and industrial emissions. The Windsor LUR model estimates were applied to each child’s home and school location. Both annual average and seasonal LUR models were developed to coincide with the timing of the personal sampling and to assess whether source contributions changed as a result of seasonal conditions such as meteorological conditions. For clarification, the LUR models were not weighted by the amount of time each child spent in different locations. The models did not include measures of meteorological variables.

Concentrations of NO_2_ in the participant’s homes, backyards, and on their person were included as a method to assess how well these frequently used proxy methods compared with our personal hopane levels. These were measured using the Ogawa passive samplers (Ogawa & Company, Pompano Beach, FL, USA) as described in Wheeler et al. [37]. These samples were deployed concurrently in all three environments on a daily basis during the personal exposure monitoring.

Finally, PM_2.5_ and NO_2_ measurements were taken at the two National Air Pollution Surveillance (NAPS) stations in Windsor, ON, Canada and metrics of road density were created. The total length of local and major roads within buffers of 100, 200, 300, 400, 500, 750, and 1000 m of participant homes were generated using ESRI ArcGIS 9.0 (Redlands, CA, USA).

### 2.4. Factors of Personal Activity and Home Characteristics

Factors of personal activity were measured using questionnaires. Daily time–activity patterns for each child were recorded using time–activity diaries (TADs), and these also tracked time spent indoors at home, outdoors at home, in transit, at school, indoors away from home, and outdoors away from home. These data were also combined with the personal PM_2.5_ monitoring data to estimate PM_2.5_ exposure attributable to time spent in transit [41].

Home characteristics were measured using several approaches. A questionnaire was administered by field technicians to collect additional data relating to housing characteristics, such as the presence or absence of an attached garage and approximate age of the home. Parents completed daily questionnaires to provide information on cooking and cleaning which are known sources of combustion-related particulate matter [37]. Indoor and outdoor PM_2.5_ were continuously measured in all homes using two DustTraks (Model 8520, TSI Incorporated, Shoreview, MN, USA) with 2.5 µm inlets to restrict measured particles to PM_2.5_. These measurements were used to estimate daily infiltration factors (*F_inf_*) and the ambient/non-ambient components of indoor particle concentrations. These analyses have been reported previously for the Windsor residences and also include estimates of the ambient and non-ambient components of the children’s personal exposures [41,46]. Daily home air exchange rates were measured using a tracer gas method [37].

### 2.5. Statistical Analyses

Statistical analyses involved testing for linear associations between hopanes and between hopanes and the aforementioned TRAP proxies along with factors of personal activity and home characteristics. Statistical methods included Pearson’s correlation coefficient and least-squared regression (LSR) models. As 14 participants were included in both seasons, data for analyses including both seasons were not independent. The sample size was insufficient to control for repeated measures across seasons; all analyses were stratified by season.

To examine the source of hopanes measured in the study, the ratio of a_b_hop and H17a was estimated and compared to those in the literature to identify coal-burning and engine-oil-sourced hopanes. [30] LRS analyses were conducted, by season, with a_b_hop and H17a as the dependent and independent variables, respectively. The ratio of a_b_hop to H17a was estimated as the slope of the regressions along with 95% confidence intervals.

The five species of hopanes were compared to factors of personal activity and home characteristics as well as all proxy methods for TRAP exposure. To begin, tests of linear associations were conducted using Pearson’s correlation coefficient. The robustness of these linear associations was further tested with LSR models with a hopane species as the dependent variable and a TRAP proxy, factor of personal activity, or home characteristic as the independent variable. LSR models were screened by testing the model assumption of normality using the Durbin–Watson test.

The “proc corr” procedure in the statistical software package SAS EG (SAS V. 9.3 within SAS 176 EG V. 5.1, SAS Institute, Cary, NC, USA) was used to estimate Pearson correlation coefficients (*r*). LSR model analyses were conducted in R. Both Research Ethics Boards for Health Canada and the University of Windsor provided approval for the study (REB-2005–0023, approved date: 15 August 2005.).

## 3. Results

### 3.1. Demographics, Personal Activity, and Personal, Indoor, and Outdoor NO_2_ Levels

Study characteristics for participants with valid personal hopane data are included in Table 1. The majority of the children included in the analyses were male, aged between 10 and 12 years, living in detached homes, with forced air heating systems and electric stoves. In summer, when school was not in session, the children spent more time at home (on average, 77% compared to 68% in winter) and more time outdoors, either at home or away from home (on average, 11% compared to 5% in winter) (Table 2). Integrated NO_2_ measurements made in the children’s homes, backyards and on their persons are presented in Table 3. In both seasons, personal NO_2_ levels were mostly associated with indoor levels (winter *r* = 0.74, summer *r* = 0.75) and weakly associated with outdoor levels (winter *r* = −0.09, summer *r* = 0.22). In both seasons, outdoor NO_2_ levels were higher than both the corresponding indoor and personal levels, which were similar. NO_2_ levels for all locations were significantly higher in winter.

### 3.2. Hopane Concentrations, Seasonality, and Ratios

**Figure 1:**
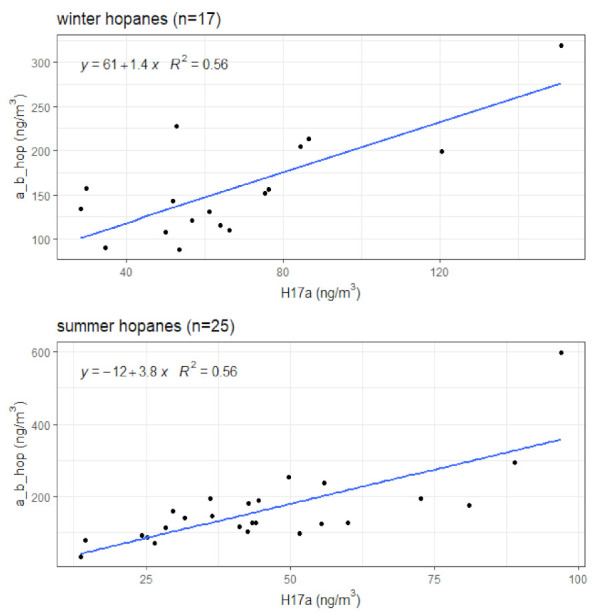
Estimation of a_b_hop: H17a ratios by season using LSR models.

Less than 1% of the individual hopane concentrations fell below the limit of detection (LOD) and these (*n* = 4) were limited to H22S and H22R, and these values were replaced using LOD/2. Winter and summer hopane levels are presented in Table 4. The summer mean value for H17a was significantly lower than the winter (*p* = 0.01) but all other hopane concentrations were similar by season. All five of the personal hopanes had moderate to strong associations with each other, ranging from *r* = 0.63–0.97 in winter and 0.60–0.97 in summer. H17a was the least related to H22S and H22R, with only moderate associations of *r* = 0.63 and 0.71 in winter and *r* = 0.66 and 0.60 in summer, respectively, see Table 4. The LSRs between a_b_hop and H17a in winter and summer are presented in Figure 1, with full models in Table S1. The ratio of a_b_hop:H17a in winter (1.4; 95%CI: 0.7–2.1) and summer (3.8; 95%CI: 2.4–5.3) suggest a lubricating engine oil source rather than being from coal emissions [30].

### 3.3. Relationships between Personal Hopane Measures and Typical TRAP Proxy Measures

**Figure 2:**
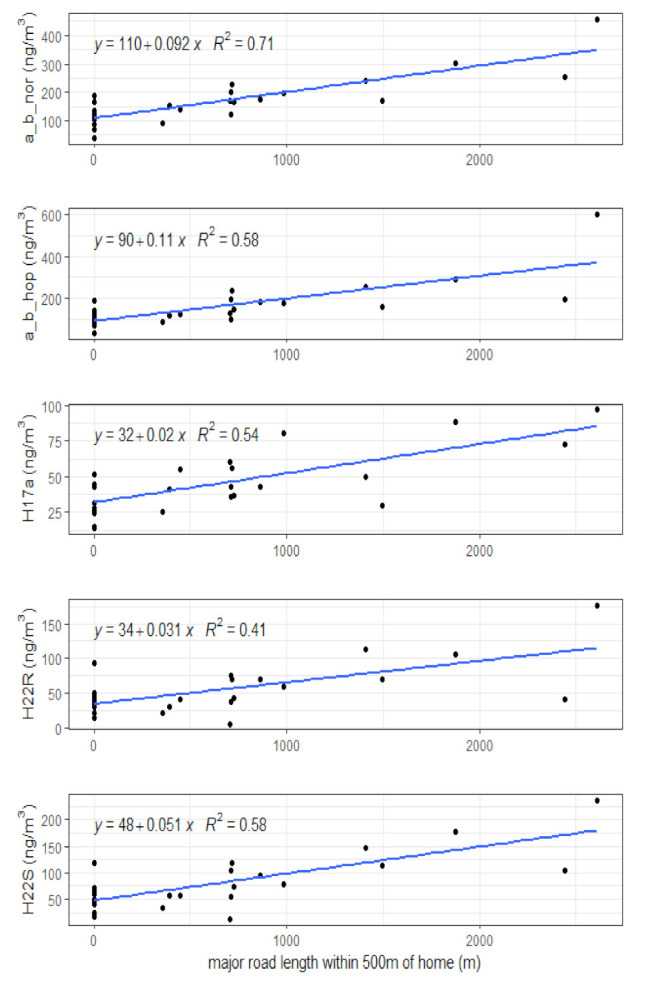
Summer hopanes: LSR models for personal summer hopanes and length of major roads within 500 m of participant homes.

Pearson correlation analyses by season are presented in Table S2. The robustness of these linear associations was further tested with LSR analyses with tests for residual normality. These final results are presented in Table S3. In summer, personal hopane measures were positively associated with metrics of major road lengths and LUR estimates. The length of major roads around the child’s residence was positively associated with hopanes for all buffers (*r* = 0.47–0.84), with the exception of H22R for the 100 and 1000m buffers. As well, LUR model estimates were associated with summer personal hopanes (*r* = 0.41–0.74). However, these LUR associations were considerably weaker than the density of major roads within several buffers. The annual NO_2_ LUR 2005 estimates were the most strongly associated with all five personal summer hopane species (*r* = 0.65–0.74) compared to any of the other LUR models (Table S2). The annual PM_2.5_ LUR 2005 estimates were also associated with each personal summer hopane (*r* = 0.46–0.68). It is likely that the associations with the LUR estimates were on account of the LUR models including major roads as predictors. In general, LUR models that predominantly included road predictors with smaller buffers sizes (<300 m) tended to better reflect the personal hopane measures. LSR analyses for summer hopanes with residual normality included only major road buffers and LUR model estimates (Table S3). Of the various buffer sizes for major roads, the 500 m buffer had the strongest relationship with hopanes. Further, for each of the five hopanes, the 500 m major road buffer had a stronger linear relationship with hopanes than the LUR estimates, with the exception of H22R (Table S3). Scatterplots and LSR for models of personal summer hopanes and the length of major roads within 500 m are presented in Figure 2.

**Figure 3:**
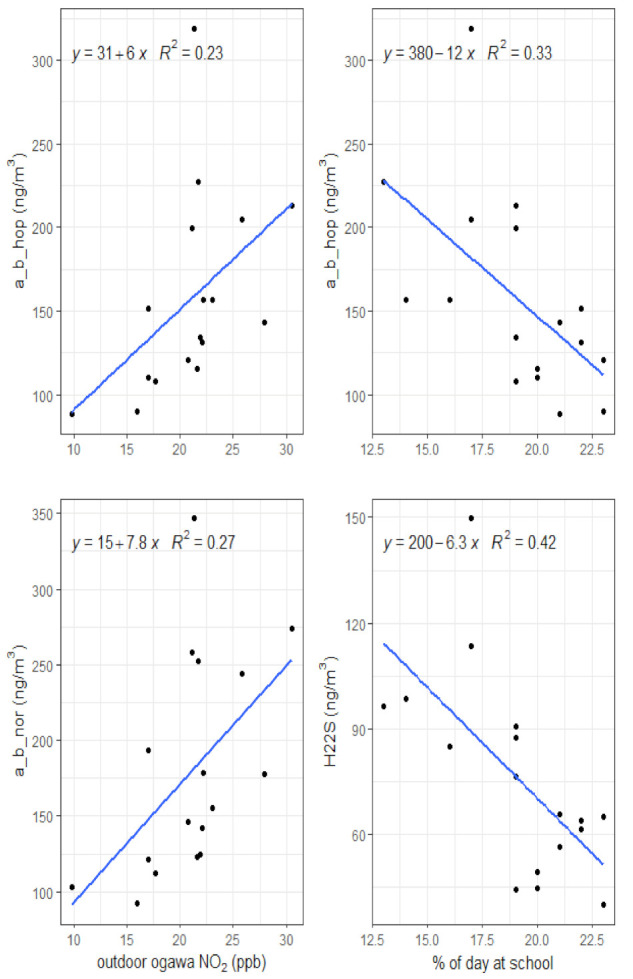
Winter hopanes: LSR models for personal winter hopanes and outdoor NO_2_ measured in participant backyards (associated with a_b_hop and a_b_nor) and percent of the day spent at school (associated with a_b_hop and H22S).

In contrast with summer, associations between personal winter hopanes and the density of major roads were not present. While Pearson’s correlations for the winter season indicated some association with the smaller buffer sizes, LSR analyses did not reveal these associations to be robust. Personal microenvironmental activity exhibited the strongest relationship with winter hopanes. Hopanes a_b_nor and H22S exposures showed a negative relationship with time spent at school (*r* = −0.57 and *r* = −0.65, respectively), with more time at school leading to lower personal hopane concentrations, which may be a result of the school locations compared to the home locations. While time spent indoors at home was associated with all hopanes, LSR model residuals were non-normal. Only time spent at school and outdoor NO_2_ concentrations measured in participant backyards demonstrated robust relationships with personal winter hopanes (Figure 3).

In summary, major road length within 500m of participant homes was the most meaningful predictor of summer hopanes. These relationships were stronger than with those of LUR models, potential reasons for this being that LUR models were optimized to estimate exposures from a range of emission sources (see Table S4), while hopanes typically originate from a single source; in this case, engine oil lubricants, as indicated by the a_b_hop:H17a ratios. This may explain why we see a weaker relationship between the personal hopanes and the LUR estimates. In winter, this strong linear association between hopanes and density of major roads in proximity to the children’s homes is not present. This may be due to the increased time spent away from home in winter (at school), which was negatively associated with personal hopanes. We investigated whether the generally weaker winter associations were due to the smaller available sample size. This was done indirectly by repeating the summer analyses using the corresponding summer children (*n* = 14), thus, balancing the sample sizes. However, these results (not shown) were consistent with the complete summertime data set, suggesting that the seasonal differences we observed are robust.

## 4. Discussion and Conclusions

We acknowledge that there is no perfect TRAP tracer, but our comparison of a range of typically applied TRAP exposure methods, with personal measures of hopanes, offers some unique insight. This study showed that GIS-related measures summarizing the density of major roads within 500 m of the children’s residence were the best surrogate of personal exposure to TRAP, as indicated by their personal hopane data. In summer, there was a tendency for major roads at greater distances away from the home to have more of an impact on personal hopanes. This was not reflected in winter, the reasons being unclear. Greater photochemical degradation of the hopanes in summer [26] would reduce the influence of more distant roads and lead to the opposite seasonal behavior. Other meteorological factors may have been involved, but additional studies would be needed to uncover the underlying causes. Another possible explanation could be that the children were active across a wider spatial area during the winter (86% at home in summer vs. 73% in winter, Table 2). Therefore, in summer, the length of major roads surrounding their home had a stronger influence on their personal hopane exposures.

LUR models are a method frequently used to assign estimates of chronic exposures to TRAP in epidemiological studies, particularly those developed to predict NO_2_. However, for all such models, their specificity to TRAP depends upon the predictors included in the individual model. This is exemplified in the Windsor results, where, in comparison with the direct measures of major roads in proximity to the child’s home, the Windsor LUR models explained considerably less of the children’s personal hopane variability. The likely reason for this is that the Windsor LUR models include a high proportion of predictors that are not directly related to traffic and included predictors, such as dwelling density, industrial sources and commercial areas [44]. We felt that it was important to evaluate typical optimized LUR models that include not just traffic predictors, as these are frequently employed for estimating exposures in epidemiological research. Results of the personal hopane correlation analyses with the LUR predictors also reflect findings by Sbihi et al. [32], whereby LUR estimates explained only 39% of the variability in hopane levels in settled dust from the same Windsor homes. Simpler LUR models that mainly consider local road network predictors would be expected to lead to an improved ability to explain personal hopane exposures. However, such models would potentially have a lower R^2^ compared to the NO_2_ observations, which would be counter to the motives of the optimized model development in most circumstances. Epidemiological studies that use NO_2_ LUR models as a proxy for exposure to TRAP should be aware of the model predictors (e.g., different sources) and, thus, their importance, before concluding that any observed associations with health outcomes are solely due to TRAP.

Evidence that variations in the children’s residential outdoor NO_2_ was not a strong proxy of TRAP exposure is also provided by the personal, indoor and outdoor NO_2_ measurements. The outdoor NO_2_ concentrations were almost double those of the personal and indoor data, suggesting that penetration into the homes of the outdoor NO_2_ was limited. If it did represent TRAP then we would expect its direct measurements, particularly the personal measurements, to correlate more strongly with personal hopane concentrations. However, very few associations were found in either season, with the only exceptions being between outdoor NO_2_ and a_b_nor and a_b_nor in the winter (Figure 3). In contrast, in both seasons, the personal NO_2_ concentrations were strongly associated with the indoor NO_2_ concentrations but not the outdoor (Table 3). This is suggestive that personal exposures to NO_2_ for this study were related more with indoor sources, such as gas cooking and heating. This may be as a result of the children spending a significant amount of their time indoors (Table 2). This does lead to question whether outdoor NO_2_ readily infiltrates into homes in the same manner as PM_2.5_, which has been shown to have a range of infiltration rates (PM_2.5_ range = 0.26–0.36) for these Windsor homes, especially the black carbon component, which hopanes would be likely to be associated with (BC range = 0.28–0.59) [46].

Several studies have highlighted the importance of location and activity patterns on individual’s exposure to TRAP [47]. Dons et al. [48,49] used detailed time–activity diaries, GPS tracking, and personal exposure monitoring and found that time–activity patterns play an important role. They found that adults spent 6% of the day in transport, which accounted for 21% of their personal exposure to black carbon, a marker of TRAP. Wu et al. [50] coupled real-time GPS tracking and adult personal exposure monitoring and found that time spent in a vehicle (on average 4.5% of their time) explained 48% of the variation in personal exposure to particle-bound polycyclic aromatic hydrocarbon concentrations. Among the children in our Windsor study, Van Ryswyk et al. [41] showed that transit exposure, while short, resulted in approximately 9% of the children’s total daily exposure to PM_2.5_. Presumably, this PM_2.5_ would be enriched in hopanes. However, we did not find that, the percent of the PM_2.5_ attributed to time in transit increased and the personal hopane levels also increased. This may be because only 9% of the daily Windsor PM_2.5_ resulted from time spent in transit, which was insufficient for influencing the personal 5-day hopane measures.

Attached garages are known to contribute to elevated personal and residential indoor levels of benzene, another marker of TRAP [51]. More frequent use of garages in winter, along with longer engine warming periods, could lead to greater indoor hopane levels via infiltration, and could have weakened the winter associations between personal hopanes and the measures of major road lengths. The diminished seasonality in our study may be a result of the large amount of time the children spent indoors. A more significant rise in temperature from outdoor to indoor in winter could lead to a greater reduction in hopanes from outdoor to indoor due to evaporation, thus, attenuating the outdoor seasonality in hopanes, as observed by the personal samples.

Some important limitations of this study should be noted. The small sample size and restricted number of monitoring events over the two seasons may limit the generalizability of our results and it prevented further stratifications and analysis. This was a result of the high costs of conducting personal exposure monitoring and the need to capture 5 days of exposure to ensure sufficient capture of measurable hopanes. It is unclear if the children’s activity patterns were influenced by the personal monitoring. We tested the data seasonally and feel that the seasonal differences are robust. However, future studies with larger sample sizes and more complete sets of indicators for heating and non-heating periods of the year would allow for a broader assessment of factors that modify or predict the hopane relationships. Another critical assumption made in this study is that hopanes have few sources beyond engine oil lubricants. We did evaluate ratios between key hopanes, as per Irei et al. [30], which provided confidence that our primary source was lubricating oil. We attempted to identify any associations with indoor sources, such as cooking, but found no significant effects. Sbihi et al. [32] also found no associations with indoor sources for hopanes in settled dust for the same Windsor homes; however, a Florida study indicated that this might not be the case for all homes. In a 2008 study, three hopanes were measured in residential indoor, residential outdoor, and other outdoor microenvironments in Tampa Bay, Florida [52]. The resulting indoor/outdoor ratio of the hopanes among nine pilot homes suggested a great deal of variability, and indoor/outdoor ratios >1 in some homes support the potential for indoor sources. However, the authors noted that the highest indoor/outdoor hopane ratios were observed when outdoor concentrations were near the limit of detection. This does suggest that infiltration for different homes could be an important component to understanding individual-level exposures to TRAP.

There is evidence that hopanes may be susceptible to photochemical degradation; however, we did not see significant seasonal differences other than for H17a, which was elevated in winter compared to summer [34,35]. However, the stronger summer associations with length of major roadways in different sized buffers around the child’s home do not suggest that photochemical degradation was an issue. Further evidence is required to understand the relationships between ambient hopanes and how they transform due to evaporation when moving from cold to warm temperatures, such as found when moving from outside to inside homes in winter.

Our results suggest that using metrics of local major road density is a useful, simple indicator of TRAP and more sophisticated estimators (LUR), or even NO_2_ measurements at the home, are not clearly better. We suggest that major road length density as an indicator is more robust to seasonal influences, whereas the seasonal differences in the more sophisticated exposure methods we evaluated need to be carefully considered when they are used for more time-resolved exposure prediction, such as for critical early life exposure windows. This suggests that attributing an association between a health outcome and NO_2_ as being due to TRAP should be made with caution. This may be especially important for any assignment of exposure to TRAP for time-dependent research, such as during children’s key growth and developmental windows, where the role of NO_2_ could vary seasonally. There is seemingly intractable heterogeneity in epidemiological research findings for pollutants, such as NO_2,_ among health studies and a leading candidate is potentially a result of differences in the various exposure assessments used, but these remain extremely difficult to isolate with currently available information. This suggests that the main sources of the exposure variability in NO_2_, whether directly measured or obtained from an LUR model, used in each epidemiological study need to be more carefully examined before concluding the results are specifically telling us something about TRAP effects (as opposed to NO_2_ effects or effects from the different urban emissions mixture containing NO_2_ or effects from combustion emissions in general). However, currently, we do not have a way to consistently judge the actual link between NO_2_ and TRAP in many of the existing health effects studies. This is a clear future need.

## Figures and Tables

**Table 1:** Study participants characteristics.

Characteristic		Winter	Summer
Gender	Female	5	9
	Male	12	16
Age	10	5	8
	11	6	10
	12	6	7
Ethnicity	Caucasian	16	23
	Other	1	2
Home Type	Detached	14	21
	Other	3	4
Heat Type	Forced Air	16	23
	Hot Water	1	2
Stove Type *	Electric	12	17
	Natural Gas	5	7
Temperature (°C)	Mean (Std Dev)	0 (3)	23(2)

* one missing value for summer.

**Table 2:** Personal per cent of time spent in different microenvironments, by season.

Season	Microenvironment	Descriptive Stats (%)				
		Mean	Std.Dev	Min	Median	Max
winter (*n* = 17)	indoors away from home	5	4	0	4	14
	indoors at home	68	5	59	68	76
	outdoors away from home	4	2	0	3	8
	outdoors at home	1	2	0	0	5
	at school	19	3	13	19	23
	in transit	4	2	1	4	6
summer (*n* = 25)	indoors away from home	9	9	0	7	27
	indoors at home	77	10	58	77	100
	outdoors away from home	5	5	0	4	19
	outdoors at home	6	5	0	5	19
	at school	0	0	0	0	0
	in transit	3	3	0	2	11

**Table 3:** Descriptive statistics and correlations of personal, indoor, and outdoor NO_2_ by season.

Season	Environment	*r*			(ppb)				
		Personal	Indoor	Outdoor	Mean	Sd	Min	Median	Max
winter (*n* = 17)	personal	1	0.74	−0.09	12.6	4.5	7.3	11.3	22.6
	indoor		1	0.06	11.1	6.8	4.5	8.7	30.8
	outdoor			1	21.0	4.8	9.8	21.6	30.5
summer (*n* = 25)	personal	1	0.75	0.22	8.0	3.3	2.8	7.0	16.8
	indoor		1	0.32	7.9	4.7	1.0	7.0	20.2
	outdoor			1	12.8	5.4	4.4	13.5	24.4

Personal and outdoor NO_2_ levels were significantly higher in winter (*p* < 0.05).

**Table 4:** Descriptive statistics and correlations of hopane species by season.

Season	Hopane	*r*					(ng/m^3^)				
		H17a	a_b_nor	a_b_hop	H22S	H22R	Mean	SD	Min	Median	Max
winter (*n* = 17)	H17a *	1	0.80	0.75	0.63	0.71	67.3	31.3	28.5	61.1	150.8
	a_b_nor		1	0.97	0.85	0.85	179.2	72.3	92.1	155.7	346.8
	a_b_hop			1	0.91	0.89	157.0	59.5	88.1	143.3	319.1
	H22S				1	0.88	75.7	28.7	39.9	65.6	149.8
	H22R					1	50.3	20.3	19.4	49.4	102.8
summer (*n* = 25)	H17a *	1	0.85	0.75	0.66	0.60	45.5	21.5	13.7	42.7	96.9
	a_b_nor		1	0.96	0.91	0.86	169.5	85.1	39.9	164.9	457.1
	a_b_hop			1.00	0.92	0.92	161.2	109.5	32.4	126.8	599.6
	H22S				1	0.97	80.9	51.7	12.7	68.9	236.2
	H22R					1	54.7	37.3	5.6	43.0	176.8

* Hopane H17a was significantly higher in winter (*p* = 0.01).

## Data Availability

The data are available upon written request to the corresponding author.

## Supplementary Materials

The following are available online at https://www.mdpi.com/article/10.3390/toxics10060284/s1, Table S1: Estimation of ratios of a_b_hop and H17a hopanes by season with LSR; Table S2: Pearson correlation coefficients (*r*) between personal exposures to hopanes and typical TRAP exposure indicators, by season; Table S3: Univariate regression models for personal hopanes in summer and winter; Table S4: Windsor LUR data.
