# Supplementary material for: An Investigation into Which Methods Best Explain Children’s Exposure to Traffic-Related Air Pollution
Source: Toxics. 2022 May 26;10(6):284. doi: 10.3390/toxics10060284 (PMC9229918; doi:10.3390/toxics10060284)
Supplement: Supplementary file 1 [file toxics-10-00284-s001.zip › toxics-1736599-supplementary Table S4/toxics-1736599-supplementary Table S1-Table S3.pdf]

toxics

MDPI

Supplementary Materials: An Investigation into which Methods Best Explain Children's Exposure to Traffic-Related Air Pollution

Keith Van Ryswyk, Amanda J. Wheeler, Alice Grgicak-Mannion, Xiaohong Xu, Jason Curran, Gianni Caravaggio, Ajae Hall, Penny MacDonald and Jeffrey R. Brook

Table S1. Estimation of ratios of a_b_hop and H17a hopanes by season with LSR.

season	intercept				slope				R^2	Durbin-Watson *p*-value
	est	LCI	UCI	*p*-value	est	LCI	UCI	*p*-value		
summer	−12.15	−84.95	60.65	0.7331	3.81	2.361	5.266	0	0.54	0.47
winter	60.81	9.8	111.83	0.0226	1.43	0.739	2.122	0.0005	0.54	0.4
a_b_hop as dependent										

Table S2. Pearson correlation coefficients *(r)* between personal exposures to hopanes and typical TRAP exposure indicators, by season.

TRAP exposure indicator	winter (*n* = 17)					summer (*n* = 25)				
	H17a	a_b nor	a_b hop	H22S	H22R	H17a	a_b nor	a_b hop	H22S	H22R
major road buffers										
100 m	0.63	0.50	0.59	0.61	0.56	0.53	0.59	0.53	0.47	-
200 m	0.56	-	0.51	0.55	-	0.57	0.68	0.65	0.60	0.53
300 m	0.56	-	0.53	0.54	-	0.65	0.73	0.68	0.66	0.57
400 m	0.53	-	-	-	-	0.70	0.82	0.75	0.75	0.65
500 m	-	-	-	-	-	0.74	0.84	0.76	0.76	0.64
750 m	-	-	-	-	-	0.80	0.78	0.68	0.62	0.50
1000 m	-	-	-	-	-	0.80	0.68	0.57	0.52	-
Home characteristics										
garage? (yes)	-	-	-	-	-	-	-	-	-	-
Attached garage	-	-	-	-	-	-	-	-	-	-
Used for parking?	-	-	-	-	-	-	-	-	-	-
how many cars in garage?	-	-	-	-	-	-	-	-	-	-
air conditioning (yes)	-	-	0.50	-	-	-	-	-	-	-
infiltration	0.49	-	-	-	-	-	0.40	-	0.41	-
personal infiltration estimates	-	-	-	-	-	-	-	-	-	-
air exchange rate	-	-	-	-	-	-	-	0.41	0.40	-
Other exposure data sources										
Central site (NAPS) $PM_{2.5}$	-	-	-	-	-	-	-	-	-	-
Central site (NAPS) NO_2	-	-	-	-	-	-0.42	-	-	-	-

Central site (NAPS) NO_x	-	-	-	-	-	-	-	-	-	-
Indoor NO_2	-	-	-	-	0.49	-	-	-	-	-
Outdoor NO_2	-	0.52	0.48	-	-	-	-	-	-	-
Personal NO_2	-	-	-	-	-	-	-	-	-	-
Sum of 'in transit' $PM_{2.5}$	-	-	-	0.59	-	-	-	-	-	-
Time spent:										
at school	-	-	-0.57	-0.65	-	-	-	-	-	-
in transit	-	-	-	-	-	-	-	-	-	-
indoors at home	-	0.61	0.66	0.62	0.66	-	-	-	-	-
indoors away from home	-	-	-	-	-	-	-	-	-	-
outdoors at home	-	-	-	-	-	-	-	-	-	-
outdoors away from home	-	-	-	-	-	-	-	-	-	-
LUR estimates										
2004 NO_2 seasonal#	-	-	-	-	-	-	0.41	0.41	0.44	0.42
2005 NO_2 seasonal	-	-	-	-	-	-	-	-	0.45	0.47
2006 NO_2 seasonal	-	-	-	-	-	-	0.58	0.52	0.65	0.54
2005 Toluene seasonal	-	-	-	-	-	-	0.44	0.59	0.57	0.72
2004 Benzene annual	0.63	0.50	0.53	0.51	-	-	0.62	0.58	0.57	0.48
2005 NO_2 annual	-	-	-	-	-	0.65	0.74	0.70	0.72	0.67
2005 $PM_{2.5}$ annual	-	-	-	-	-	0.46	0.60	0.63	0.65	0.68
2006 $PM_{2.5}$ seasonal	−0.63	−0.70	−0.72	−0.58	-	−0.44	-	-	-	-

'-' = p-value > 0.05.

# Corresponding winter and summer LUR data were used.

Table S3. Univariate regression models for personal hopanes in summer and winter.

season	hopane	predictor type	predictor	units	intercept				slope				R^2	Durbin–Watson
					est	LCI	UCI	*p*–value	est	LCI	UCI	p–value		*p*–value
summer	a_b_hop	major roads	length of major roads within 500 m of home	meters	90.5	50.8	130.1	0.0001	0.1	0.1	0.1	0	0.56	0.93
		LUR	2006 NO_2 seasonal LUR Estimate	ppb	−28.9	−176.3	118.5	0.6879	20.8	5.7	36	0.0091	0.24	0.64
			2004 Toluene annual LUR Estimate	μg/m³	−62.1	−268.4	144.3	0.5383	73	7.5	138.5	0.0306	0.17	0.86
			2004 Toluene seasonal LUR Estimate	μg/m³	−61.5	−203.4	80.4	0.3786	37	14.8	59.1	0.0022	0.32	0.85
	a_b_nor	major roads	length of major roads within 500 m of home	meters	108.7	83	134.4	0	0.1	0.1	0.1	0	0.7	0.71
		LUR	2004 Benzene annual LUR Estimate	μg/m³	−153.5	−342.6	35.6	0.1062	431.9	184	679.8	0.0016	0.36	0.94
			2004 NO_2 annual LUR Estimate	ppb	−289.9	−484.8	−95.1	0.0055	33	19.2	46.8	0.0001	0.52	0.91
			2006 NO_2 seasonal LUR Estimate	ppb	8.4	−98.3	115.1	0.8717	17.7	6.8	28.7	0.0028	0.31	0.69
			2004 $PM_{2.5}$ annual LUR Estimate	μg/m³	−605.3	−1073.5	−137.1	0.0138	49.9	19.9	79.9	0.0023	0.33	0.96
	H17a	major roads	length of major roads within 500 m of home	meters	32	23.9	40.2	0	0	0	0	0	0.52	0.4
		LUR	2004 NO_2 annual LUR Estimate	ppb	−58.6	−114.7	−2.6	0.0413	7.5	3.5	11.5	0.0008	0.4	0.58
			2004 $PM_{2.5}$ annual LUR Estimate	μg/m³	−104.9	−239.1	29.4	0.1191	9.7	1.1	18.3	0.0285	0.17	0.99
	H22R	major roads	length of major roads within 500 m of home	meters	34.4	18.4	50.4	0.0002	0	0	0	0.0005	0.39	0.68
		LUR	2004 NO_2 annual LUR Estimate	ppb	−128.5	−219.8	−37.1	0.0081	13	6.6	19.5	0.0004	0.43	0.2
			2004 NO_2 seasonal LUR Estimate	ppb	23.3	−7.4	54	0.1301	3.1	0.5	5.7	0.0197	0.19	0.79
			2006 NO_2 seasonal LUR Estimate	ppb	−13.5	−63	36	0.5776	7.5	2.4	12.5	0.006	0.26	0.63
			2004 $PM_{2.5}$ annual LUR Estimate	μg/m³	−325.1	−509.6	−140.6	0.0014	24.3	12.5	36.2	0.0003	0.44	0.52
			2004 Toluene annual LUR Estimate	μg/m³	−36.7	−99.7	26.2	0.2387	29.4	9.4	49.4	0.0059	0.28	0.64
			2004 Toluene seasonal LUR Estimate	μg/m³	−38	−79.9	4	0.0736	15.3	8.7	21.8	0.0001	0.49	0.86
	H22S	major roads	length of major roads within 500 m of home	meters	47.6	28.8	66.5	0	0.1	0	0.1	0	0.56	0.7
		LUR	2004 Benzene annual LUR Estimate	μg/m³	−100.9	−220.3	18.6	0.0936	241.1	84.5	397.6	0.0043	0.3	0.6
			2004 NO_2 annual LUR Estimate	ppb	−191.6	−312.6	−70.5	0.0035	19.5	10.9	28	0.0001	0.49	0.1
			2004 NO_2 seasonal LUR Estimate	ppb	40.3	−2.6	83.1	0.0643	4.1	0.5	7.7	0.0274	0.17	0.82

			2006 NO_2 seasonal LUR Estimate	ppb	−31.3	−93	30.3	0.3035	12.3	5.9	18.6	0.0006	0.4	0.96
			2004 $PM_{2.5}$ annual LUR Estimate	μg/m^3	−427.2	−696.2	−158.3	0.0034	32.6	15.4	49.9	0.0008	0.4	0.88
			2004 Toluene seasonal LUR Estimate	μg/m^3	−20.1	−88	47.9	0.5465	16.8	6.2	27.4	0.0034	0.3	0.71
winter	a_b_hop	NO_2	Outdoor Ogawa NO_2 average	ppb	30.5	−99.2	160.3	0.6233	6	0	12.1	0.0504	0.18	0.24
	a_b_hop	time activity	Percent time spent at school	%	379.2	202.3	556.1	0.0004	−11.6	−20.8	−2.5	0.0163	0.28	0.5
	a_b_nor	NO_2	Outdoor Ogawa NO_2 average	ppb	14.7	−139.3	168.7	0.8414	7.8	0.7	15	0.034	0.22	0.21
	H22S	time activity	Percent time spent at school	%	196.3	116.9	275.8	0.0001	−6.3	−10.4	−2.2	0.0052	0.38	0.5
